# It’s time to reward the gift of life

**DOI:** 10.15171/jnp.2016.16

**Published:** 2016-05-02

**Authors:** Bahar Bastani

**Affiliations:** Division of Nephrology, Saint Louis University School of Medicine, Saint Louis, USA

**Keywords:** Kidney transplantation, Organ shortage, End-stage renal disease, Kidney donation

**Implication for health policy/practice/research/medical education:**

Increasing supply of kidneys, decreasing transplant wait time, reducing wait list, decreasingmorbidity and mortality in end stage renal failure patients, decreasing cost to society.

April is Donate Life Month and the number of patients waiting for kidneys has reached over 100000 in the United States. Only around 16% of those in the transplant wait list can hope to get a transplant this year. The rest continue to suffer on dialysis, and on average 12 or more die every day. If we count people who die on dialysis who could be on the list but aren’t, the number is closer to 25. Every year around 15% of the patients on the waiting list are removed because they are either too sick to be transplanted or have died (1).

That so many people die each year in the United States. is a tragedy; but that so many die needlessly is a scandal. The simple fact is, an insufficient number of kidneys are donated each year to meet the demand because the National Organ Transplant Act (NOTA), adopted in 1984, prohibits providing compensation to kidney donors except under very limited conditions that aren’t of help to most potential donors.

There were good reasons at the time for congress to pass that law. First, it was largely a reaction to a misguided proposal in 1983 by Dr. Barry Jacobs that would have imported poor people from third-world countries to be organ donors in the United States, a measure that highlighted natural concerns about exploitation (2). Second, it was believed at the time that all of our transplant needs could be solved with cadaver organs if we only put together a more efficient way of retrieving, storing, and distributing organs.

After the US Congress banned compensating donors and established the Organ Procurement and Transplant Network, our combined efforts brought the annual average of kidney donations in the United States from cadavers to between 10000 and 11000 and those from living donors to about 6000. However, these numbers have remained stagnant in United States since 2005, while the number of people in need of a kidney has exponentially increased. So 85% of our demand for kidneys is unmet in the United States (1,3), and it would still be unmet even if everyone in the country signed an organ donor card, because only fewer than one percent of those who die each year do so under conditions that allow a successful transplant (4).

We can and do encourage living kidney donation, but it will never be sufficient under current policies. In the United States a third of all transplant kidneys come from altruistic donors such as friends, relatives, or the occasional non-directed donor, such as those heroic individuals who donate to set off a transplant pair exchange chain. But such altruistic donation is largely confined to those who have the time and resources that enable them to afford such generosity.

Those who lack well-off friends or connections are relegated to dialysis, which is not a real alternative. Dialysis cleans only 10% of the blood’s impurities compared to kidneys, so those relegated to a waiting list are slowly poisoned by their own body’s waste products as they wait. And, because of the law in the United States, this burden is heaviest on the poor and middle class.

As our problem in the United States gets worse, there is one country – Iran – that has solved the kidney shortage through a system of compensated donation. In some regions, there is actually a list of people waiting to donate.

While the United States and the rest of the world emphasized developing their cadaver organ networks, Iran did the opposite. It was part of the European organ network, but due to our imposition of economic sanctions, Iran was cut off from the rest of the world and relied instead on perfecting its living donor networks (3,5-7), while at the same time (although at a slower pace) improved its cadaveric transplant program.

A system of using NGOs (non-profit non-governmental organizations) to match donors and recipients evolved over the past 30 years in an effort to ensure that all who need a kidney can get one without exploiting donors. Although there was a necessary learning curve, with inevitable missteps along the way, the medical community and in turn the government took many significant steps to improve its donor compensation system to the point where in some cities the system works enviably well.

There were a number of key developments in this evolution:

- The government licensed charities to arrange kidney matches. These NGOs are staffed by volunteers who themselves are mostly transplant recipients or their close relatives, and have come to understand that the only way to keep more donors and recipients coming is to make sure the needs of both are met.
- For donors this means fair, legally binding contracts, health insurance for themselves and in some cases for their families as well, and access to the same charitable services available for transplant recipients—services such as, help with paperwork, healthcare, dental care, eye care, job services, education, small business loans and more.
- For recipients this means donors have to be screened carefully and that the immunosuppressant drugs that transplant recipients need lifelong are heavily subsidized by the government.
- The system is closed to foreigners, making it easier to do follow-up and provide consistent services, as well as keeping the expected monetary financial reward affordable for the locals.
- Drug users are banned from donating – this the Iranian medical community did far too late in the history of its program, but doing so is standard practice today.
- Donors see psychologists and/or social workers to make sure they have thoroughly thought through all aspects of donation.
- Because the NGOs treat donors and recipients equally, there is no sense that either side is exploiting the other. Both donors and recipients see themselves as working together with the NGO to improve their lives.

Understandably, it is difficult for US policymakers to admit that Iran may be doing something right. But the point is not that we should become like Iran, but only to acknowledge that we may learn something from that country’s experience. To ignore that Iran has succeed at solving a problem that costs tens of thousands of American lives every year just because of our political differences is willful blindness.

Enough American kidney disease patients have died. It’s time to open our minds and learn from Iran.

## Author’s contribution

BB is the sole author of the paper.

## Conflicts of interest

None.

## Funding/Support

None.
